# A Case of Pancreatic Ductal Adenocarcinoma in an Elderly Adult With Heterotaxy Syndrome

**DOI:** 10.7759/cureus.63664

**Published:** 2024-07-02

**Authors:** Zhongqian Lin, Aruni Rahman, Samuel Quintero

**Affiliations:** 1 Internal Medicine, NewYork-Presbyterian (NYP) Brooklyn Methodist Hospital, Brooklyn, USA; 2 Gastroenterology, NewYork-Presbyterian (NYP) Brooklyn Methodist Hospital, Brooklyn, USA

**Keywords:** invasive pancreatic tumor, pancreatic atrophy, polyspenia, ductal pancreatic adenocarcinoma, heterotaxy syndrome (hs)

## Abstract

Heterotaxy is a syndrome characterized by a spectrum of anatomical anomalies in organ lateralization due to embryological errors. It frequently involves intrathoracic organs, especially the heart, leading to congenital abnormalities. Abdominal organs can also be affected, causing clinical features such as sepsis from asplenia or intestinal volvulus; however, these are less studied. Currently, there is no data on the relationship between heterotaxy and malignancy. We present an interesting case of an elderly adult admitted for a workup of newly diagnosed pancreatic ductal carcinoma, who was found to have heterotaxy of the stomach and spleen, with eventual tumor invasion of these organs. This case suggests that heterotaxy may increase the risk of gastrointestinal malignancy and result in a poorer prognosis due to the complexity of tumor resection involving additional organs.

## Introduction

Heterotaxy is a rare finding and is defined as an anatomical variant of intrathoracic and abdominal organs from the typical arrangement, caused by disruption of left-right axis orientation during embryonic development [1,2]. It is most commonly associated with congenital cardiac malformations but can also involve abdominal organs such as the spleen, stomach, and liver [3]. Current data regarding non-cardiac heterotaxy are very limited, including its association with GI malignancy, although case reports do mention a few cases of atrophied pancreas in patients with heterotaxy. We present a case of an elderly adult with pancreatic ductal adenocarcinoma who was found to have heterotaxy of the stomach and spleen.

## Case presentation

**Figure 1:**
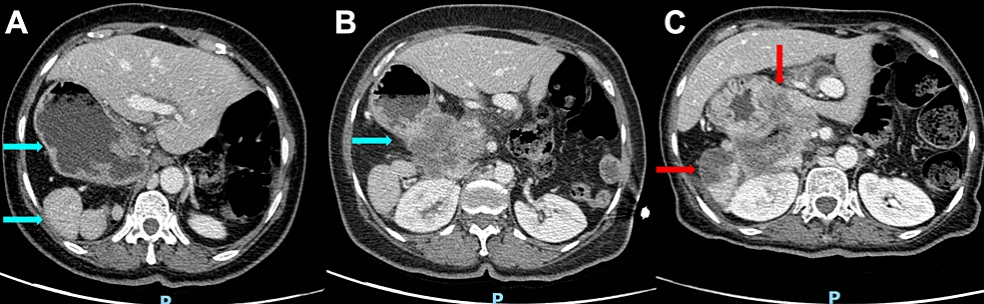
(A) Initial contrast-enhanced CT scan of the abdomen and pelvis (CTAP) revealing the stomach and three splenic tissues located in the right upper quadrant (RUQ). (B) Initial CTAP showing a pancreatic head mass abutting the lesser curvature of the stomach. (C) CTAP four months later showing the previous pancreatic head mass increased in size with new invasion of the spleen in the RUQ.

**Figure 2:**
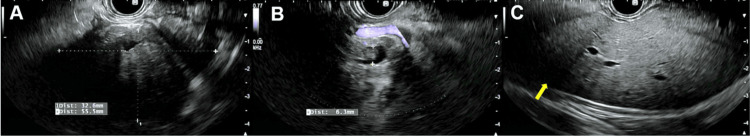
Endoscopic ultrasound (EUS): (A) Pancreatic body mass, (B) Dilation of pancreatic duct, (C) Atrophic pancreatic tail.

**Figure 3:**
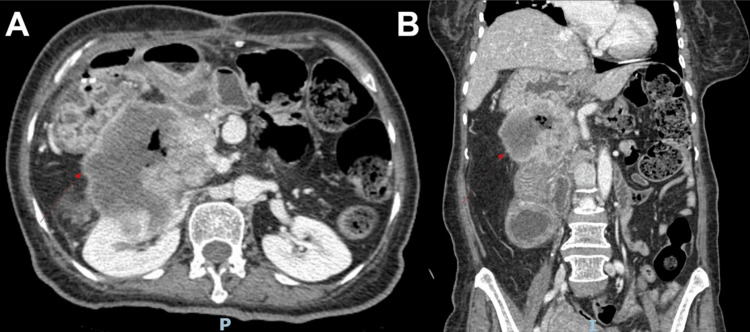
CT scan of the abdomen and pelvis revealed disease progression: (A) Axial view, (B) Coronal view.

A 69-year-old female with a past medical history of type-2 diabetes mellitus and a recent finding of a mass in the pancreatic head from an outpatient CT scan of the abdomen and pelvis (CTAP) a month ago, without further workup, presented with progressively worsening generalized abdominal pain of four months' duration. She was subsequently admitted for symptom management and malignancy workup. Physical examination revealed generalized abdominal tenderness, most prominently in the epigastric region, but no alarming features were noted. Initial vital signs and routine blood work were unremarkable. CTAP with intravenous contrast showed a 5.3 x 6.3 x 6.7 cm hypodense pancreatic mass centered in the pancreatic head and uncinate process, abutting the right margin of the superior mesenteric artery, right kidney, right adrenal gland, proximal duodenum, jejunum, and lesser curvature of the stomach, with loss of the intervening fat plane between the mass and these organs. Interestingly, the stomach was found to be in the right upper quadrant (RUQ) with loss of a discernible fat plane and associated mural thickening, as well as polysplenia in the RUQ (Figure 1). Upon obtaining baseline past records, a CTAP from 2 years ago already showed stomach and spleen tissues in the RUQ and severe pancreatic atrophy. While inpatient, endoscopic ultrasound (EUS) with biopsy demonstrated 6 mm pancreatic ductal dilatation and confirmed the diagnosis of pancreatic ductal adenocarcinoma, as well as visualization of pancreatic tail atrophy (Figure 2). She was discharged and completed two cycles of the FOLFIRINOX chemotherapy regimen in the anticipation of downsizing the tumor for possible future resection. However, the patient’s functional status worsened, and she had numerous hospital visits for intractable abdominal pain and small bowel obstruction. She was eventually enrolled in a hospice program. Four months after the first visit, the patient was admitted again for poorly controlled cancer pain in the abdomen and transitioned to inpatient hospice. On the last admission, a repeated CTAP revealed a pancreas mass that was 9.0 x 8.1 cm x 7.6 cm in size with central necrosis, complete circumferential encasement of the proximal duodenum with fistulization, representing further worsening of previously known organ invasions, and new invasion to the spleen in the RUQ likely due to the failure of chemotherapy (Figure 3).

## Discussion

Most studies of heterotaxy syndrome are related to congenital cardiac abnormalities such as isomerism of atrial appendages in neonatal populations with a high morbidity and mortality rate [4]. For example, right atrial isomerism results in bilateral right atria and atrial appendages. Patients with this condition often present with lesions such as anomalous pulmonary venous drainage, leading to cyanosis and right-to-left shunting. In contrast, left-sided isomerism typically presents later in childhood and is associated with less severe forms of congenital heart disease [5]. For abdominal organs, the spleen, liver, stomach, and intestinal tract are reported to be in abnormal positions with potential clinical features such as intestinal volvulus [6]. Although not universally present, heterotaxy is frequently associated with either polysplenia, occurring in approximately 90% of affected patients, or asplenia. Polysplenia is often linked with cardiac or biliary malformations, while asplenia significantly increases the risk of sepsis [7]. In this case, the patient had incidental and asymptomatic findings of heterotaxy of the stomach and spleen without cardiac abnormality, likely the reason she remained relatively healthy until this age.

Mentions of the association between heterotaxy syndrome and malignancy are limited primarily to a few case reports, with one specifically for pancreatic cancer. Interestingly, all reported malignancies were located within the hepatobiliary system. For instance, one case detailed successful pancreatectomy for cancer of the terminal part of the common bile duct in a patient with disorientation of the pancreaticoduodenal complex [8]. Another case involved a young adult male in his twenties with left atrial isomerism and dextrocardia who developed multifocal metastatic hepatocellular carcinoma [9]. More intriguing and similar to our case was an 82-year-old female with heterotaxy involving the small bowel on the RUQ, polysplenia on the left, and a complete right-sided pancreas, who was diagnosed with pancreatic cancer and subsequently underwent successful surgical resection despite complex anatomy [10]. In contrast, our patient presented with densely packed right-sided anatomy, with the tumor invading the stomach and multiple splenic tissues on the right side, rendering resection difficult and risky. Overall, the presence of additional organs such as polysplenia complicates tumor resection and contributes to a poorer prognosis.

There are case reports of patients with heterotaxy and polysplenia concurrently exhibiting agenesis of the dorsal pancreas, although pancreatic malignancy has not been explicitly documented in these cases [11]. While pancreatic atrophy is known to precede pancreatic cancer in the general population, tumors typically originate from the same location as the atrophic changes [12]. In our case, the tumor is located in the pancreatic head and uncinate process, whereas the baseline atrophy was observed in the tail region. Based on this case, there is biological plausibility that patients with abdominal heterotaxy may face an increased risk of gastrointestinal and hepatobiliary malignancies, particularly pancreatic cancer, compared to the general population, highlighting the need for further research in this area.

Lastly, our patient presented with findings of heterotaxy and an atrophic pancreas years before the development of malignancy, yet had not undergone screening as there are no current guidelines for this condition. Similarly, in the aforementioned case of hepatocellular carcinoma, surveillance for liver disease and hepatocellular carcinoma was recommended. Given the potential association between malignancy and heterotaxy syndrome, there is an argument for investigating guidelines for cancer screening in these patients, such as routine CT imaging.

## Conclusions

In conclusion, due to the uncommon occurrence of heterotaxy syndrome, there is limited data available, particularly concerning abdominal organs. This case suggests a possible association between heterotaxy syndrome and cancer, with potentially worse complications for the patient due to the proximity of extra organs, which may impede surgical resection compared to the general population. Therefore, there is a compelling need for further research to establish the association between heterotaxy syndrome and malignancy. Such studies could enhance medical knowledge and potentially inform the development of screening guidelines if a positive correlation is confirmed.
